# Evolution of eukaryotic single-stranded DNA viruses of the *Bidnaviridae* family from genes of four other groups of widely different viruses

**DOI:** 10.1038/srep05347

**Published:** 2014-06-18

**Authors:** Mart Krupovic, Eugene V. Koonin

**Affiliations:** 1 Institut Pasteur, Unité Biologie Moléculaire du Gène chez les Extrêmophiles, Department of Microbiology, Paris 75015, France; 2 National Center for Biotechnology Information, National Library of Medicine, National Institutes of Health, Bethesda, MD 20894, USA

## Abstract

Single-stranded (ss)DNA viruses are extremely widespread, infect diverse hosts from all three domains of life and include important pathogens. Most ssDNA viruses possess small genomes that replicate by the rolling-circle-like mechanism initiated by a distinct virus-encoded endonuclease. However, viruses of the family *Bidnaviridae*, instead of the endonuclease, encode a protein-primed type B DNA polymerase (PolB) and hence break this pattern. We investigated the provenance of all bidnavirus genes and uncover an unexpected turbulent evolutionary history of these unique viruses. Our analysis strongly suggests that bidnaviruses evolved from a parvovirus ancestor from which they inherit a jelly-roll capsid protein and a superfamily 3 helicase. The radiation of bidnaviruses from parvoviruses was probably triggered by integration of the ancestral parvovirus genome into a large virus-derived DNA transposon of the Polinton (polintovirus) family resulting in the acquisition of the polintovirus PolB gene along with terminal inverted repeats. Bidnavirus genes for a receptor-binding protein and a potential novel antiviral defense modulator are derived from dsRNA viruses (*Reoviridae*) and dsDNA viruses (*Baculoviridae*), respectively. The unusual evolutionary history of bidnaviruses emphasizes the key role of horizontal gene transfer, sometimes between viruses with completely different genomes but occupying the same niche, in the emergence of new viral types.

Viruses with single-stranded (ss)DNA genomes constitute a large class of economically, medically and ecologically important pathogens that infect hosts in all three domains of life^1^. Recent metagenomic studies have shown that these viruses are extremely widespread in various environments and are highly diverse genetically^2^,^3^,^4^. However, the understanding of the patterns of evolution of these viruses remains incomplete. Generally, a growing body of evidence supports a network-like mode of viral genome evolution, whereby constituent functional modules are acquired from different sources and are occasionally replaced with functional equivalents, apparently in response to changing ecological conditions^1^,^5^,^6^,^7^,^8^. Any organism with which a virus shares the niche—be it the host, co-infecting unrelated virus or any other mobile genetic element, such as plasmid or transposon—can serve as the source of new genes and functional modules. Viruses with large double-stranded (ds)DNA genomes display high genomic plasticity and are particularly prone to horizontal gene exchange^8^,^9^,^10^. By contrast, acquisition of new genetic material by smaller viruses with single-stranded genomes appears to meet various constraints, including difficulty to accommodate larger amounts of extra genetic information due to the inherent limitations of the small internal capsid volume and pressure for genome compactness as well as selection to preserve functional secondary structure elements within their genomes^11^. Indeed, although not unprecedented^12^,^13^,^14^, horizontal acquisition of non-homologous genes by eukaryotic viruses with small ssDNA genomes appears to be generally rare, despite the high rates of recombination among these viruses^15^.

Eukaryotic ssDNA viruses typically have small icosahedral capsids and for genome replication rely on the rolling circle-like mechanism initiated by a distinct virus-encoded endonuclease^1^,^16^. Unexpectedly, however, viruses of the recently established family *Bidnaviridae* do not conform to this general pattern. The family *Bidnaviridae* includes small isometric viruses that infect the silkworm *Bombyx mori*^17^. The genome of these viruses consists of two linear ssDNA segments of 6.5 (VD1) and 6 (VD2) kilobases (kb) which are packed into separate capsids. The complete genome sequences are currently available for three closely related isolates of the *Bombyx mori* bidensovirus (BmBDV). Based on the linear structure of the genome, BmBDV was initially placed in the family *Parvoviridae*, but upon closer examination it was reclassified into an independent virus family^17^. Indeed, unlike any other known ssDNA virus, instead of the typical rolling circle replication initiation endonuclease (RCRE), BmBDV encodes a type B DNA polymerase^18^ and has been suggested to replicate its genome via the protein-primed mechanism reminiscent of that characterized in adenoviruses^19^. The virions of bidnaviruses are superficially similar to those of parvoviruses^17^ although no homolog of the parvovirus capsid protein (CP) has been recognized. By contrast, one of the experimentally identified structural proteins of BmBDV displays sequence similarity to the minor CP of reoviruses which have dsRNA genomes^20^. These observations suggest that horizontal gene transfer might have played an important role in the evolution of bidnaviruses. However, the puzzling evolutionary relationships between BmBDV and other viruses have not been investigated thus far. Here, we report an in depth analysis of the provenance of all bidnavirus genes and describe the evolutionary trajectories that led to the emergence of this unique viral group.

## Results

### Bidnaviruses evolved from parvoviruses

**Figure 1:**
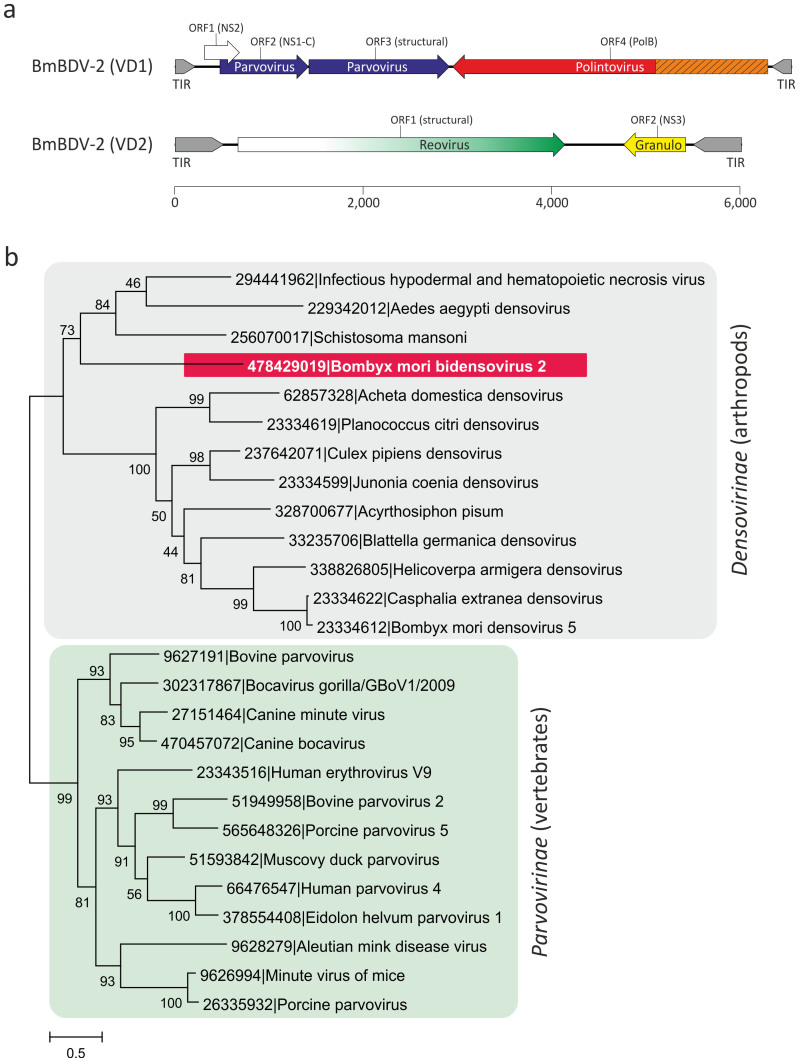
(a). Genome map of *Bombyx mori* bidensovirus 2 (BmBDV-2). Terminal inverted repeats (TIR) are shown in grey. BmBDV-2 genes are shown with arrows indicating the direction of transcription. The genes are colored according to their deduced origin: ancestral parvovirus genes are shown in blue, genes derived from polintoviruses, reoviruses, and granuloviruses are colored red, green, and yellow, respectively. The region of the PolB gene encoding the potential terminal protein implicated in the protein-primed DNA replication is shown in orange and hatched. The gene for nonstructural protein 2 (NS2), for which provenance remains unclear, is depicted with an open arrow. (b). Maximum likelihood tree of the S3H domains from parvoviral and bidensoviral NS1 proteins. The tree was rooted on the branch between the two subfamilies of the *Parvoviridae*, *Densovirinae* and *Parvovirinae*. GenBank identifiers for all analyzed taxa are provided next to their names. Numbers at the branch points represent SH-like local support values.

The single-strandedness of the bidnavirus genomes as well as the dimensions and morphology of the virions seem to point to their possible relatedness to parvoviruses. Another feature shared between these two groups of viruses is the superfamily 3 helicase (S3H^21^). In parvoviruses, the S3H domain is found at the C-terminus of the conserved non-structural protein 1 (NS1) whereas in BmBDV the S3H is encoded by the VD1 ORF2 as a stand-alone protein (Fig. 1a). However, the inverted terminal repeats in bidnaviruses do not form hairpins characteristic of parvoviruses^19^,^22^ and the N-terminal RCRE domain of the parvoviral NS1 is lacking. Furthermore, the major structural protein of BmBDV encoded by VD1 ORF3^18^,^23^ has not been shown to be related to the typical parvoviral CPs. Consequently, the direct evolutionary link between bidnaviruses and parvoviruses remains an open question. To establish a framework for investigating the bidnavirus evolution, we first assessed their connection to the members of the *Parvoviridae*.

Based on the genomic features as well as the host range of corresponding viruses, the family *Parvoviridae* is divided into two subfamilies: members of the *Parvovirinae* infect vertebrate hosts, whereas members of the *Densovirinae* replicate in arthropods^24^. We first analyzed the relationship between the product of BmBDV VD1 ORF2 and the S3H domains of the parvoviral NS1 proteins. Alignment of the BmBDV VD1 ORF2 protein with the corresponding S3H domains of the NS1 proteins from a range of the *Parvoviridae* representatives infecting different hosts revealed the conservation of all three diagnostic motifs typical of S3H proteins^21^, consistent with the previous report^25^. Maximum likelihood analysis of the S3H domains firmly places BmBDV among the arthropod-infecting viruses within the *Densovirinae* subfamily, suggesting that BmBDV either evolved from an ancestor belonging to this viral group or horizontally acquired the S3H gene from a densovirus.

**Figure 2:**
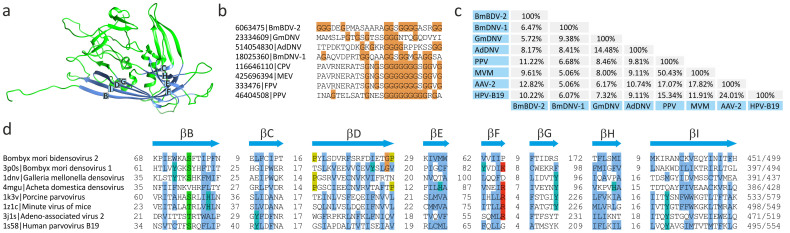
Analysis of the major capsid protein of BmBDV. (a). Ribbon diagram of the X-ray structure of the capsid protein of *Bombyx mori* densovirus 1 (PDB ID: 3P0S)^27^. The core jelly-roll domain is highlighted in blue with the β-strands constituting the two β-sheets, BIDG and CHEF, indicated with corresponding letters. (b). Sequence alignment of the glycine-rich regions found in the N-termini of the parvoviral and bidnaviral capsid proteins. (c). Pairwise identity values between the sequences of capsid proteins of BmBDV and parvoviruses for which high-resolution structures are available. The accession numbers for the compared proteins are the same as in panel (d). (d). Multiple sequence alignment of the BmBDV and parvoviral capsid protein regions corresponding to the 8 β-strands forming the jelly-roll fold. The secondary structure elements of the BmBDV capsid protein were predicted using PSI-Pred^57^, whereas those of the parvoviral proteins were determined experimentally. Parvoviral sequences are indicated with their PDB accession numbers. Abbreviations: BmBDV-2, *Bombyx mori* bidensovirus 2; GmDNV, *Galleria mellonella* densovirus; AdDNV, *Acheta domestica* densovirus; BmDNV-1, *Bombyx mori* densovirus 1; CPV, canine parvovirus; MEV, mink enteritis virus; FPV, feline panleukopenia virus; PPV, porcine parvovirus; MVM, minute virus of mice; AAV-2, adeno-associated virus 2; HPV-B19, human parvovirus B19.

The virions of parvoviruses for which high resolution structural information is available, consist of homologous CPs that adopt the jelly-roll fold^26^,^27^,^28^,^29^. This fold is formed by two antiparallel β-sheets each consisting of four β-strands, BIDG and CHEF, respectively (Fig. 2a^30^). No homolog of the parvovirus CP has been thus far identified in BmBDV. However, given that the pairwise sequence identity between homologous parvoviral CPs is often about 5%^28^, the failure to establish the relationship between the CPs of BmBDV and other parvoviruses using standard BLAST search is not surprising.

The major constituent protein of the BmBDV virions is encoded by VD1 ORF3^18^,^23^. We noticed that similar to the CPs of both vertebrate- and arthropod-infecting parvoviruses^31^, the product of VD1 ORF3 contains an N-terminal glycine-rich region (Fig. 2b), suggesting that this protein might be homologous to the CPs of parvoviruses. We compared the sequence of VD1 ORF3 with the parvoviral CPs, for which the X-ray structures have been solved, using PROMALS3D^32^, which aligns the protein sequences taking into account their predicted (for the target sequence) or experimentally determined secondary structure elements. This analysis showed that although the overall sequence identity between CPs of BmBDV and parvoviruses is low (generally below 13%), it is in the range of similarity observed between CPs of distantly related parvoviruses (Fig. 2c), consistent with previous reports^28^. Importantly, in addition to the glycine-rich region (Fig. 2b), the BmBDV CP encompasses counterparts to all 8 β-strands that form the core of the jelly-roll fold (Fig. 2a), and moreover, these predicted β-strands are located at positions equivalent to those in the parvoviral CPs (Fig. 2d). Thus, we conclude that, the absence of significant sequence similarity notwithstanding, the major CP of BmBDV is a homolog of the parvoviral CPs.

At the N-terminus of the CP (preceding the glycine-rich and the jelly-roll domains), most parvoviruses contain a conserved phospholipase A2 domain which is important for virus entry^33^,^34^. This domain is not present in the CP of BmBDV; however, biochemical characterization of the BmBDV identified a *B. mori*-encoded lipase associated with the virions^23^. Thus, unlike parvoviruses, bidnaviruses appear to package a functionally equivalent host enzyme that is presumably required for efficient cell entry. The linear structure of the bidnavirus genomes and the conservation of the parvoviral-like S3H and CP proteins strongly suggest that bidnaviruses originated from a parvovirus ancestor.

### ‘Polintoviruses' at the origin of *Bidnaviridae*

**Figure 3:**
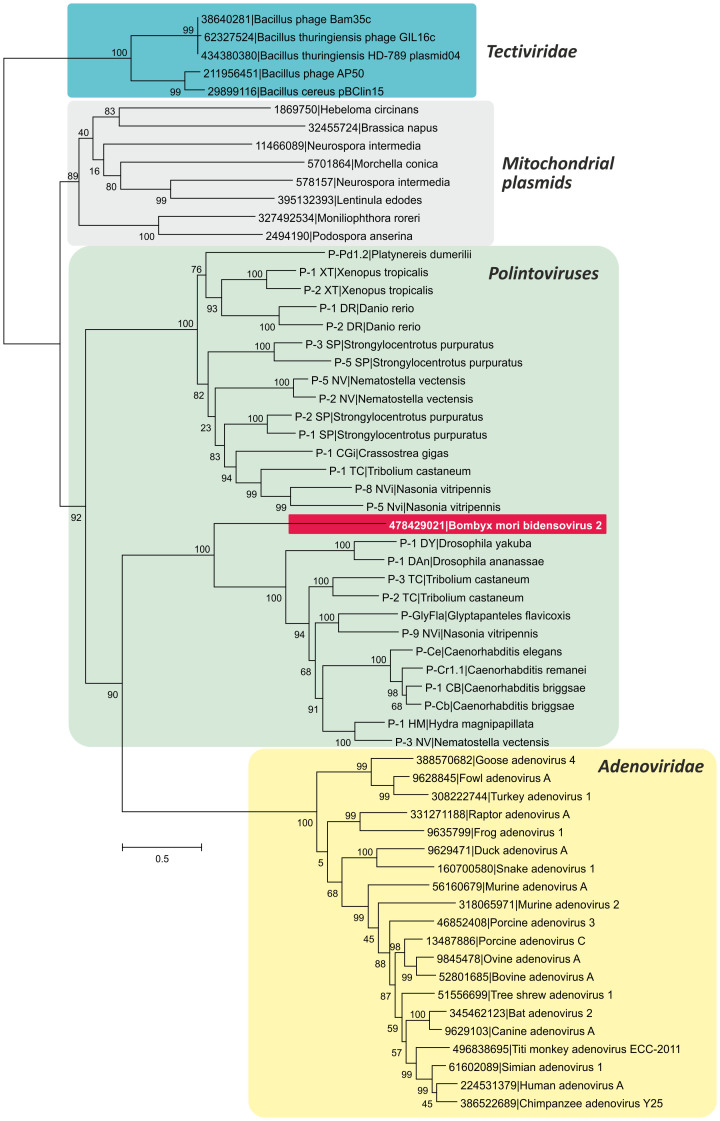
Maximum likelihood tree of the PolB proteins from diverse viruses and plasmids. Sequences of polintoviruses were retrieved from the Repbase Update database^58^ as described in Methods. The tree was rooted on the branch of bacterial tectiviruses. Numbers at the branch points represent SH-like local support values.

The main feature distinguishing bidnaviruses from all other ssDNA viruses, including parvoviruses, is the presence of a gene encoding the protein-primed DNA polymerase of the family B (PolB). We hypothesized that the key event that led to the secession of the bidnaviruses from parvoviruses was the acquisition of the PolB gene. Consequently, identification of the likely donor of the PolB gene is central for understanding the origin of the *Bidnaviridae*. A BlastP search against the non-redundant (nr) NCBI protein database seeded with the sequence of PolB of BmBDV returned as the best hit a PolB from the endoparasitoid wasp *Glyptapanteles flavicoxis* (ACE75264; E = 6e-79). Analysis of the genomic context of the *G.* *flavicoxis* PolB gene showed that it is an integral part of the previously described large transposable element^35^ of the Polinton/Maverick family^36^,^37^. We have recently demonstrated that these transposons are derived from viruses which we provisionally denoted ‘Polintoviruses'^38^. Polintoviruses, like bidnaviruses, possess long inverted terminal repeats and have been suggested to replicate via a single-stranded intermediate by a protein-primed mechanism^36^. Notably, an identical sequence (5′-GTGTGTGT-3′) is found at the tips of the inverted terminal repeats in both BmBDV and the polintovirus from *G.* *flavicoxis*. To further investigate the provenance of the bidnavirus PolB, we constructed a maximum likelihood tree (Fig. 3), which included protein-primed PolB sequences from various mobile elements, such as polintoviruses, adenoviruses, linear mitochondrial plasmids, and bacterial tectiviruses. These four types of elements form four well-supported clades, with the BmBDV PolB firmly nested within the polintovirus clade (Fig. 3). This tree topology strongly suggests that the bidnavirus PolB is of polintovirus origin.

Viruses that replicate their genomes via the protein-primed mechanism typically encode terminal proteins which are covalently attached to the genome termini and serve as primers for DNA replication^39^. No such gene has been identified in the bidnavirus genomes thus far. Previous biochemical characterization of the BmBDV virions identified a minor component of ca. 53 kDa encoded by the PolB gene which has been suggested to correspond to a C-terminally truncated BmBDV PolB^40^. We compared the PolB protein of bidnaviruses with those of viruses encoding dedicated terminal proteins, such as adenoviruses and tectiviruses, and identified a unique N-terminal domain of ca. 400 amino acids in BmBDV protein (Fig. 1). A similar N-terminal domain is also present in polintoviral PolBs and shares some conserved motifs with the bidnavirus PolB N-terminal sequence (Fig. S1). Thus, the N-terminal domains of BmBDV and polintoviral PolBs might correspond to the terminal proteins involved in the initiation of the protein-primed genome replication of other replicons, as previously proposed in the case of polintoviruses^36^. Notably, in bacteriophages (tectiviruses) and adenoviruses terminal proteins are encoded immediately upstream of the PolB gene^39^, suggesting that in bidnaviruses and polintoviruses the two genes are fused, as is also the case in linear eukaryotic plasmids^41^. Given the extreme sequence divergence of the viral terminal proteins, the lack of detectable sequence similarity between the putative terminal proteins of bidnaviruses/polintoviruses and any other proteins is not surprising.

### Acquisition of the minor capsid protein from reoviruses

**Figure 4:**
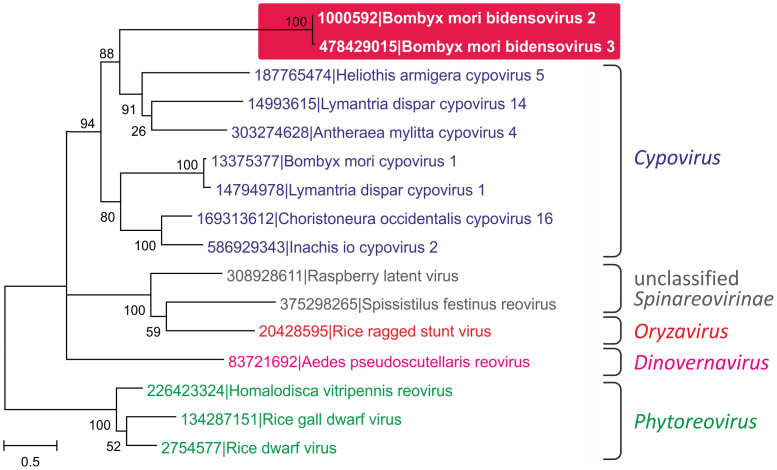
Maximum likelihood tree of the minor capsid proteins of reoviruses. Different genera of the family *Reoviridae* are indicated. The tree was rooted on the branch between the taxa belonging to two different *Reoviridae* subfamilies, *Sedoreovirinae* and *Spinareovirinae*. Numbers at the branch points represent SH-like local support values.

The second BmBDV gene encoding a structural protein (besides VD1 ORFs 3) is located on the second genomic segment, VD2. The VD2 ORF1 encodes the 133 kDa minor capsid protein (1160 amino acids) that shows sequence similarity to proteins of some reoviruses^20^. Gene transfer between viruses with RNA and DNA genomes is not common; however, several such cases have been inferred^12^,^14^,^42^ and might have played a major role in the origin and evolution of ssDNA viruses^1^,^43^,^44^. To better understand the function and evolution of the VD2 ORF1, we analyzed its sequence using PSI-Blast. Searches against the nr protein database showed that VD2 ORF1 of BmBDV shares with diverse reoviruses the C-terminal domain of ca. 570 amino acids, whereas the N-terminal regions do not appear to be homologous. After the first PSI-Blast iteration, only sequences from cypoviruses (subfamily *Spinareovirinae*) were retrieved (best Blast hit to a protein from *Choristoneura occidentalis* cypovirus 16; ACA53381, E = 2e-06). However, after several iterations, proteins of reoviruses from other genera were retrieved as well, including the extensively studied rice dwarf virus (genus *Phytoreovirus*, subfamily *Sedoreovirinae*). The homologous regions of reoviral and bidnaviral proteins were aligned and a phylogenetic tree was constructed using the maximum likelihood method (Fig. 4). In this phylogeny, reoviral sequences formed clades compatible with the current viral taxonomy^45^. The bidnaviruses cluster with members of the genus *Cypovirus*, which infect arthropods. Given the shared host range, it appears most likely that VD2 ORF1 was acquired horizontally by a bidnavirus ancestor from a co-infecting cypovirus. In reoviruses, VD2 ORF1 homologs are found in the outer capsid shell and are involved in the host recognition and virus entry^46^,^47^, suggesting a similar function for the VD2 ORF1 protein of BmBDV.

### Gene shuffling among arthropod viruses with ssDNA and dsDNA genomes

A non-structural protein encoded by VD2 ORF2, denoted NS3, has been reported to share sequence similarity with NS3 proteins from several densoviruses^48^ and *Cryptophlebia leucotreta* granulovirus (genus *Betabaculovirus*, family *Baculoviridae*)^49^. The NS3 homolog from *Junonia coenia* densovirus is essential for viral DNA replication^50^, suggesting that BmBDV protein might also play an important function during the viral life cycle.

**Figure 5:**
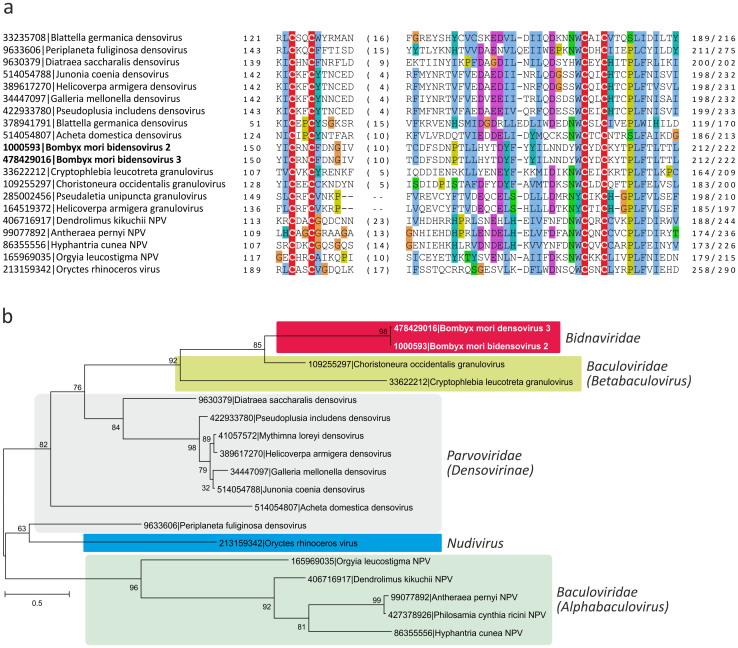
Analysis of the bidnaviral NS3 proteins. (a). Multiple sequence alignment of the bidnaviral NS3 proteins with the homologs from diverse arthropod viruses. The four conserved cysteine residues comprising the Zn-binding domain are highlighted with the red background. (b). Maximum likelihood tree of the NS3 proteins. Viruses belonging to different taxonomic groups (indicated on the right) are highlighted with different colors. Numbers at the branch points represent SH-like local support values.

We performed a search for BmBDV NS3 homologs using PSI-Blast and found that this protein is distributed much wider than currently considered^17^. In addition to homologs previously identified in densoviruses and *Cryptophlebia leucotreta* granulovirus, other viruses with dsDNA genomes from the family *Baculoviridae* (genera *Alphabaculovirus* and *Betabaculovirus*) and the unassigned genus *Nudivirus* were also found to encode NS3-like proteins (Fig. 5a). The function of the NS3-like proteins in dsDNA viruses is not known either. Lange and Jehle have previously suggested that NS3-coding genes could have been transferred between dsDNA baculoviruses and ssDNA parvoviruses^49^; however, the exact evolutionary history of these genes as well as the directionality of such putative gene exchange has not been assessed. Phylogenetic analysis of the NS3-like proteins revealed a complicated patter. In this tree, BmBDV did not cluster with densoviruses as in the case of NS1 (Fig. 1b). Instead, it formed a well-supported clade with granuloviruses (genus *Betabaculovirus*). Consistently, in BlastP analysis, the best match was to the protein gp025 (YP_654446; E = 5e-28) from *Choristoneura occidentalis* granulovirus, whereas the best-matching homolog among densoviruses (*Galleria mellonella* densovirus, NP_899649) was retrieved with a substantially lower significance (E = 5e-05). Unexpectedly, densoviruses themselves did not form a monophyletic clade (Fig. 5b): *Periplaneta fuliginosa* densovirus clustered with the *Oryctes rhinoceros* virus (genus *Nudivirus*) as a sister clade to the alphabaculoviruses. This tree topology is best consistent with multiple cases of horizontal gene transfer among arthropod-infecting viruses with ssDNA and dsDNA genomes. In particular, BmBDV most likely acquired its NS3 gene from a granulovirus rather than from a densovirus.

Sequence alignment of the NS3-like proteins from diverse viruses revealed a conserved Zn-finger motif (Fig. 5a). Proteins with Zn-finger motifs have been previously shown to function as inhibitors of apoptosis, known as IAP proteins, in numerous baculoviruses^51^. We hypothesize that NS3-like proteins might represent a novel family of apoptosis inhibitors in arthropod-infecting viruses. The observation of sporadic distribution and conservation of NS3-like proteins in widely different viruses that replicate in related arthropod hosts is consistent with this hypothesis.

## Discussion

**Figure 6:**
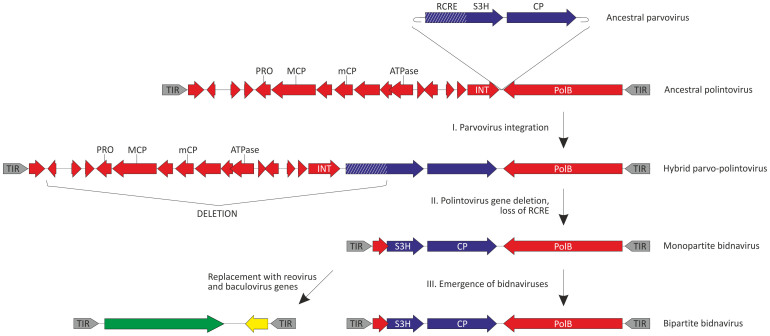
An evolutionary scenario for the origin of bidnaviruses. Parvoviral and polintoviral genes are shown in blue and red, respectively. The small red gene next to the TIR does not show significant sequence similarity between bidnaviruses and polintoviruses but, given the similar size and genomic location, could nevertheless have been derived from a polintovirus. Reoviral and baculoviral genes are colored green and yellow, respectively. TIR, terminal inverted repeats; PRO, adenovirus-like cysteine protease; MCP, major capsid protein; mCP, minor capsid protein; ATPase, genome packaging ATPase; INT, retrovirus-like integrase. See text for the details.

In this work we have investigated the origin and evolution of bidnaviruses, a unique group of ssDNA viruses. We demonstrate that the major structural protein of BmBDV is homologous to the typical jelly-roll capsid proteins of parvoviruses (Fig. 2). Furthermore, phylogenetic analysis of the NS1-like S3H domains firmly places BmBDV within the densoviruses (Fig. 1b). These results combined with the linear genome structure strongly suggest that bidnaviruses originate from the *Parvoviridae*. Although it is not currently possible to pinpoint the exact ancestral parvovirus, it appears reasonable to suggest that the split postdates the separation of vertebrate and arthropod-infecting parvoviruses. The key event in the emergence of the *Bidnaviridae* family apparently was the loss of the characteristic RCRE domain of parvoviral NS1 proteins, which could have been concomitant or subsequent to the acquisition of the PolB gene from an arthropod-associated polintoviruses (Fig. 3). Perhaps the most parsimonious evolutionary scenario includes transfer of ancestral parvoviral genes into the polintovirus scaffold (Fig. 6). The fact that parvoviruses often integrate into the host genomes^52^,^53^ lends credence to this hypothesis. Such directionality of events would explain the origin of the genomic termini of bidnaviruses which are distinct from those of known parvoviruses but are similar to the termini of polintoviruses. Polintoviruses are typically 15–20 kb-long and encode a set of genes implicated in viral capsid assembly, maturation and genome packaging, as well as some uncharacterized genes^38^. Under the present evolutionary scenario, these genes were selectively lost from the intermediate parvovirus-polintovirus hybrid until a genome sufficiently compact to be accommodated within the parvovirus-like capsid evolved. Given the conservation of the terminal structure, it appears most likely that the second, VD2-like, segment of BmBDV evolved directly from the main segment encoding the CP and PolB by replacement of the parvoviral and polintoviral genes with genes from reoviruses and baculovuses (Fig. 6). Thus, in an unexpected turn, bidnaviruses might be seen as a derivative of polintoviruses in which the virion morphogenetic unit is replaced by that of parvoviruses. It seems pertinent that some polintoviruses contain integrated gypsy-like retrotransposons^54^ whereas polintoviruses themselves occasionally integrate into the genomes of larger polydnaviruses^35^,^54^. These cases of viral and transposon chimerism remind of a Russian-doll, whereby genomes of smaller viruses integrate into those of the larger ones.

The alternative scenario involving emergence of bidnaviruses from a parvoviral ancestor which acquired *polB* gene and TIRs from polintoviruses via horizontal gene transfer appears less likely due to the following considerations. First, such gene transfer would require at least two separate, low-frequency illegitimate recombination events to occur within the short genome of the ancestral parvovirus, one for the introduction of the ‘left' TIR and the other for the transfer of the ‘right' TIR along with the *polB* gene. Furthermore, both recombination events would have to be simultaneous because single recombination would produce a replication-deficient entity. Second, the introduction of the TIRs and *polB* gene into the parvovirus scaffold would have to be immediately compensated by the loss of non-essential ancestral genes in order to produce a genome sufficiently compact to be incorporated into parvovirus-sized virions.Furthermore, the emerging recombinant virus would have to be competitive in order not to be removed from the population.Such urgency is not required under the first scenario because a parvoviral ancestor integrated, via the RCRE-mediated mechanism, into the polintovirus scaffold would have ample opportunities to evolve within the context of the host chromosome until sufficient fitness was achieved.

The subsequent events in the evolution of bidnaviruses include acquisition of novel genes from dsRNA reoviruses and dsDNA granuloviruses (Fig. 4 and 5). Recombination between RNA and DNA viruses that apparently requires a reverse transcription step does not appear common but a similar transfer of a capsid-encoding gene from a tombusvirus (ssRNA) to a circo-like virus (ssDNA) has been recently reported^12^,^14^. Conceivably, the acquisition of the receptor-binding protein from a reovirus affected the host specificity of the ancestral bidnavirus. The NS3 gene, apparently acquired from granuloviruses (Fig. 5), might encode a representative of a novel family of proteins involved in modulation of antiviral defense in arthropods, unrelated to the IAP family proteins of baculoviruses^51^. The promiscuity of genes involved in fast-evolving virus-host interactions appears to be a general trend in the viral world, with these genes traveling over large distances between viruses with very different genome types. An obvious prerequisite for such gene exchange is the intersection of the host range of the involved viruses. Indeed, all four groups of viruses identified here as contributors to the bidnavirus evolution infect arthropod hosts. Consequently, recombination between viruses that by default are considered to be unrelated molds new viral genomes with novel properties, as is the case with the bidnaviruses.

## Methods

The genome sequences of *Bombyx mori* bidensovirus 2 have been downloaded from GenBank using accession numbers AB033596 (segment VD1) and S78547 (segment VD2). Homologues of the bidensoviral proteins were searched for using PSI-BLAST^55^. Distant homology detection was also performed using HHpred^56^; however, no additional homologues other than those detected using PSI-Blast could be identified. Highly similar sequences were removed using Blastclust at http://toolkit.tuebingen.mpg.de/blastclust. Multiple sequences alignments were built using Promals3D^32^. Sequence identities were calculated using SIAS at http://imed.med.ucm.es/Tools/sias.html. The secondary structure was predicted using PSI-Pred^57^. The polintovirus PolB sequences were recovered from Polinton nucleotide sequences, which were downloaded from the Repbase Update database^58^, as described previously^38^. For phylogenetic analysis, gapped columns (more than 30% of gaps) and columns with low information content were removed from the alignment^59^. Maximum likelihood analysis was carried out by using PhyML 3.1 (REF. 60), with the WAG model of amino acid substitution, including a gamma law with 4 substitution rate categories, and an estimated proportion of invariable sites.

## Author Contributions

M.K. collected the data; M.K. and E.V.K. analyzed the data and wrote the manuscript. Both authors read and approved the final version.

## Supplementary Material

Supplementary Information Figure S1
