# Supplementary material for: Evolution of eukaryotic single-stranded DNA viruses of the Bidnaviridae family from genes of four other groups of widely different viruses
Source: Sci Rep. 2014 Jun 18;4:5347. doi: 10.1038/srep05347 (PMC4061559; doi:10.1038/srep05347)
Supplement: Supplementary Information — Figure S1 [file srep05347-s1.pdf]

Evolution of eukaryotic single-stranded DNA viruses of the *Bidnaviridae* family from genes of four other groups of widely different viruses

Mart Krupovic[a*] and Eugene V. Koonin[b]

[a] Institut Pasteur, Unité Biologie Moléculaire du Gène chez les Extrêmophiles, Department of Microbiology, Paris 75015, France

[b] National Center for Biotechnology Information, National Library of Medicine, National Institutes of Health, Bethesda, MD 20894, USA

* - corresponding author

E-mail: krupovic@pasteur.fr

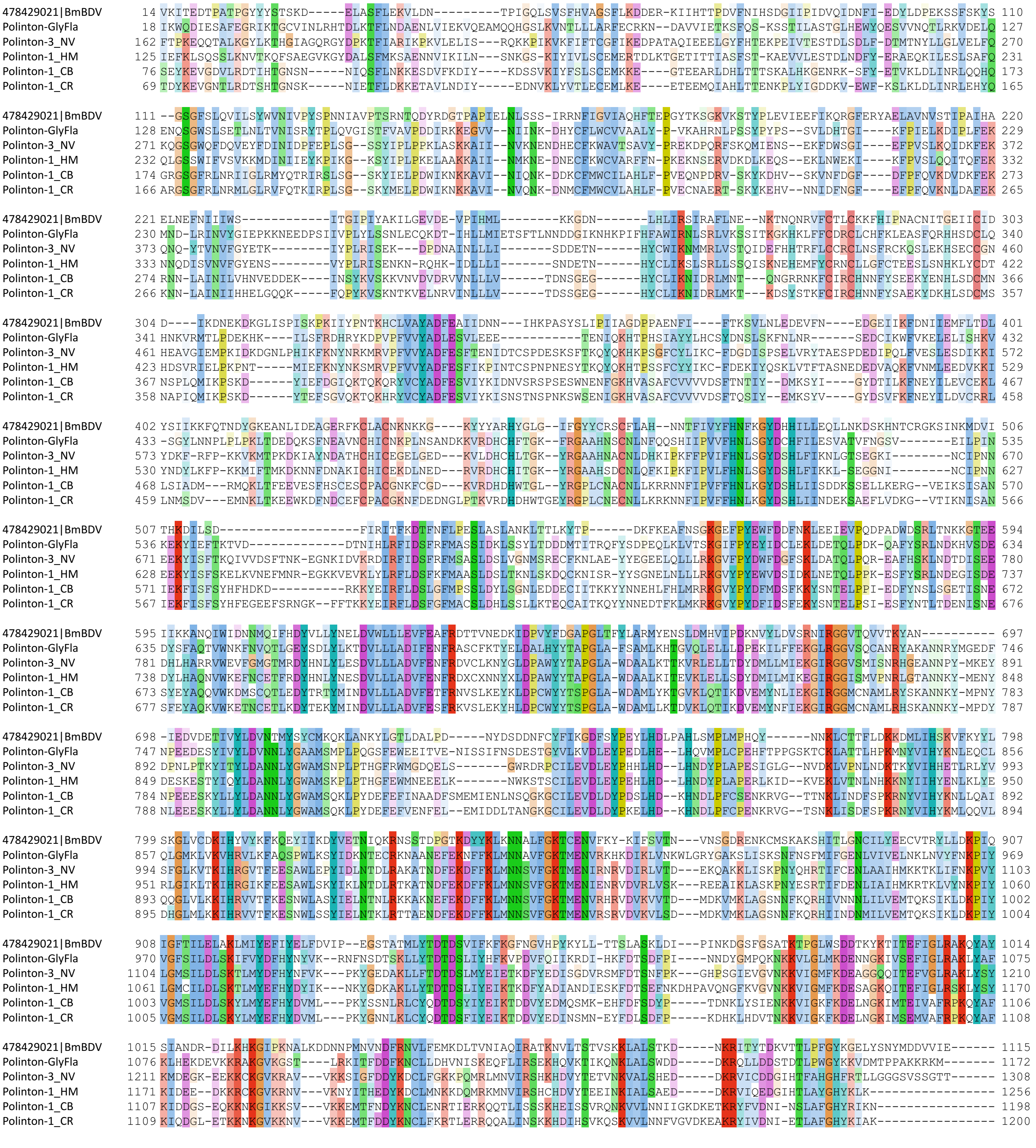

Figure S1. Multiple sequence alignment of the type B DNA polymerases from BmBDV and selected polintoviruses.
